# Sensitive detection and differentiation of rhinoviruses and enteroviruses by a nested real-time RT-PCR assay

**DOI:** 10.1128/spectrum.02203-25

**Published:** 2026-02-09

**Authors:** Masahiro Ogura, Takuma Ohnishi, Mizuki Yaginuma, Hisato Kobayashi, Munehiro Furuichi, Rika Inose, Shingo Kato, Masayoshi Shinjoh

**Affiliations:** 1 Hanah-MediTech, Co., Ltd., Tokyo, Japan; 2 Department of Pediatrics, Keio University School of Medicine, Tokyo, Japan; 3 Clinical Laboratory, Keio University Hospital 34787, Tokyo, Japan; University of Brescia, Brescia, Italy

**Keywords:** rhinovirus, enterovirus, discrimination, real-time PCR, dual infection

## Abstract

Rhinoviruses (RVs) and enteroviruses (EVs) are important respiratory pathogens. Although numerous molecular assays have been developed for detection of RVs and EVs, their genetic similarities pose challenges for molecular differentiation. In this study, we described a real-time nested reverse transcription (RT)-PCR assay using SYBR green and RV- and EV-specific reverse primers to differentially detect RVs and EVs. The primers were designed so that the numbers and locations of mismatches should be the most adequate for objective viruses and the least adequate for opposite viruses using all EV and RV sequences in GenBank. The assay was validated using nasopharyngeal swab specimens from pediatric patients who have fever and/or respiratory symptoms at Keio University Hospital from November 2021 to January 2023 and tested positive for Human Rhinovirus/Enterovirus by the FilmArray Respiratory Panel 2.1. The species and serotypes were identified by analyzing sequences of PCR products using the BLAST program. The results of the present RT-PCR assay and the BLAST analysis were completely consistent with each other. Furthermore, the current assay revealed the cases of dual infections of RV and EV. No significant differences were observed in patient demographics or clinical courses among viral species. The assay presented here may be the most suitable for routine diagnosis and surveillance of RV and EV infections.

**IMPORTANCE:**

We describe a real-time nested reverse transcription-PCR assay that enables us to differentially detect rhinoviruses and enteroviruses. Differential diagnosis of rhinovirus and enterovirus infections has not been succeeded because of their genetic diversities and similarities. We resolved this problem by using specific PCR primers that were designed by in silico analysis of all rhinovirus and enterovirus sequences obtained from GenBank. The developed method was validated by applying to more than 100 nasopharyngeal swab specimens from pediatric patients in Keio University Hospital in Japan and analyzing with the BLAST algorithm. The assay may be suitable for routine diagnosis and surveillance of rhinovirus and enterovirus infections.

## INTRODUCTION

Rhinoviruses (RVs) are the most frequent etiologic agents of respiratory tract infections, with clinical manifestations, including otitis media, croup, bronchiolitis, and pneumonia, and exacerbations of underlying chronic lung diseases, such as asthma in children (1–6). RVs are non-enveloped, positive-sense, single-stranded RNA viruses belonging to the genus *Enterovirus* of the family *Picornaviridae*. The genus *Enterovirus* includes 12 enterovirus species (A through L), which are referred to hereafter as enteroviruses (EVs), and three rhinovirus species (A, B, and C). There are currently 84 serotypes for rhinovirus A, 30 for B, and 56 for C (7). EVs cause a wide variety of diseases in infants and young children, including hand, foot, and mouth disease (HFMD), encephalitis, aseptic meningitis, myocarditis, conjunctivitis, and respiratory and gastrointestinal tract diseases (8). Certain diseases are associated with specific EV serotypes: enterovirus A71 and coxsackievirus A16 cause HFMD, and enterovirus D68 causes respiratory illnesses and acute flaccid myelitis (AFM) (9, 10). It has been reported that enterovirus D68 shows cross-reactivity with RVs on a respiratory molecular diagnostic platform (11).

Numerous molecular assays have been developed for detection of RVs and EVs by using reverse transcription PCR (RT-PCR) (12), nested PCR (13, 14), real-time RT-PCR (15), nucleic acid sequence-based amplification (NASBA) (16), and more recently RT loop-mediated isothermal amplification (RT-LAMP) (17), RT strand invasion-based amplification (RT-SIBA) (18), and RT recombinase polymerase amplification (RT-PRA) (19). Most of these assays target the 5ʹ noncoding region, which has highly conserved sequences among RVs and EVs. Because of extensive molecular variability and cross-similarity in these viruses, differentiation of RVs from EVs required additional procedures including electrophoresis (20), probe hybridization (13), restriction enzyme digestion (21), sequencing (22), and the use of locked nucleic acid probe (23). However, these assays, to our knowledge, have not been shown to successfully differentiate all RV and EV prototype strains.

After the onset of the coronavirus disease 2019 (COVID-19) pandemic, the multiplex PCR-based testing, such as the FilmArray Respiratory Panel 2.1 (bioMérieux, Marcy l'Étoile, France), for respiratory infections has become more frequently used in clinical settings in Japan (24–26). Since October 2020, Keio University Hospital has been implementing the FilmArray Respiratory Panel 2.1 in addition to general infection control measures to prevent the entry and clustering of COVID-19 in the pediatric ward. One defect of this panel is, however, that RVs and EVs cannot be distinguished.

In this study, we have developed a nested real-time PCR assay to differentiate RVs and EVs using PCR primers which were designed in silicon based on the GenBank database. The assay was evaluated by comparing the results to those obtained from the testing clinical specimens obtained from the FilmArray Respiratory Panel 2.1 (bioMérieux, Marcy l'Étoile, France) for samples of pediatric patients with suspected respiratory infection during the COVID-19 pandemic.

## MATERIALS AND METHODS

### Study design

Patients aged under 20 years with fever and/or respiratory symptoms who were tested with the FilmArray Respiratory Panel 2.1 (bioMérieux, Marcy-l’Etoile, France) (27) were included in the study. From November 2021 to January 2023, nasopharyngeal swab samples were collected and tested by the FilmArray, and human rhino/enterovirus positive samples were subjected to the present RT-nested PCR assay. The specimens were stored at −80°C until assayed.

### Data collection

The information collected included FilmArray results, our rhinovirus/enterovirus PCR results, patient age, gender, underlying medical problems, previous history of asthma, oxygen administration, asthma attack, and admission in the intensive care unit.

### RNA extraction

Total nucleic acid was extracted from 200 μL of nasopharyngeal specimens using the QIAamp MinElute Virus Spin Kit (QIAGEN, Hilden, Germany) according to the manufacturer’s instructions.

### Primer design

For selection of primers, we obtained 1,628 RV sequences and 5,329 EV sequences from GenBank, which have full-length viral genome, and aligned them in the 5ʹ noncoding region and VP4 region using Molecular Evolutionary Genetics Analysis version 11 (28). Then, the number of unmatched nucleotides between each primer candidate and viral sequence in their corresponding region was counted all over the 5ʹ noncoding and VP4 regions and the most specific sequences for RVs and/or EVs were selected. Primer candidates were chosen by the following criteria: the melting temperature is as close to 58°C as possible, calculated as previously described (29–31); the sequences are not self-complimentary or complimentary between a pair of primers; the incorporation of degenerate nucleotides at less than three positions was allowed to be incorporated if adequate. In this way, the most specific sequences for RVs and/or EVs were selected. Finally, sequences of the specific primers were confirmed to have no significant homology with those of human-host viruses belonging to the *Picornaviridae* family other than the Enterovirus genus. The primers thus obtained are shown in Table 1.

**TABLE 1:** Primers

Primer	Sequence	Position^*a*^	Feature
ERV1-F	5′-CCTCCGGCCCCTGAATG-3′	449–465	Forward primer in 1st PCR for both EVs and RVs
ERV1-R	5′-AAACACGGAYACCCAAAGTAGT-3′	545–566	Reverse primer in 1st PCR for both EVs and RVs
ERV2-F	5′-GCCCCTGAATGYGGCTAA-3′	455–472	Forward primer in 2nd PCR for both EVs and RVs
ERV2-RA	5′-AAGTAGTCGGTTCCGCTG-3′	534–551	Reverse primer in 2nd PCR for EVs
ERV2-RB	5′-AAGTAGTCGGTTCCGCCA-3′	534–551	Reverse primer in 2nd PCR for EVs
ERV2-R	5′-AAGTAGTCGGTYCTRTCC-3′	534–551	Reverse primer in 2nd PCR for RVs

^*a*^ Shown by nucleotide numbers of rhinovirus A1 strain ATCC VR-1559.

### Nested RT-PCR and sequencing

The first RT-PCR was performed to amplify DNA of RVs and EVs in a 20-μL reaction volume containing 0.2 μM each of the primers ERV1-F and ERV1-R, 4 mM MgCl_2_, 0.2 mM each dNTP, 0.04 μL of RNasin Ribonuclease Inhibitor (Promega, Madison, WI), 0.04 μL of SuperScript III Reverse Transcriptase (Invitrogen, ThermoFisher Scientific, Waltham, MA), 0.08 μL of Platinum Taq DNA Polymerase (Invitrogen, ThermoFisher Scientific), 2 μL of 10 × PCR buffer for Platinum Taq, and 4 μL of extracted sample by using the GeneAmp PCR System 9700 (Applied Biosystems, ThermoFisher Scientific). The thermal cycling program consisted of 56°C for 10 min, 94°C for 2 min, and 30 three-step cycles of 94°C for 5 s, 64°C for 10 s, and 72°C for 15 s, followed by 72°C for 1 min and left at 4°C. The second PCR was performed using 2 μL of the first PCR product to detect DNA of RVs with the primers ERV2-F and RV-2R or to detect DNA of EVs with the primers REV2-F and EV2-RA/B (EV-2RA and EV-2RB were used simultaneously) by using the CFX Connect Real-Time PCR System (Bio-Rad, Hercules, CA). The condition of the second PCR was the same as that of the first RT-PCR except with 0.16 μL of 1000 × diluted Lonza SYBR Green I (Lonza, Tokyo, Japan), without ribonuclease inhibitor and reverse transcriptase, and 25 thermal cycles instead of 30 cycles. Samples were considered positive if the fluorescence curves showed exponential increase crossing the default threshold. Following DNA extraction of the second PCR product using the QIAquick PCR Purification Kit, sequencing was performed at Eurofins Genomics Inc. (Tokyo, Japan).

### Statistical analysis

Patient characteristics were summarized as frequencies and percentages for categorical variables and as medians and interquartile ranges for continuous variables. For comparing rhinovirus types, the Fischer’s exact test was used for categorical variables and the Mann-Whitney U test was used for continuous variables. The level of significance (*P*-value) was set at *P* < 0.05. Statistical analyses were conducted in R statistical software version 4.0.5 (The R Foundation for Statistical Computing).

## RESULTS

### FilmArray

During the study period, 365 nasopharyngeal swab samples underwent the FilmArray Respiratory Panel 2.1 examination. Among them, 230 samples (63.0%) tested positive. Among the positive samples, 139 (60.4%) were positive for human rhino/enterovirus. Of these, 115 samples were subjected to verification of the present RT-nested PCR assay. The remaining 15 samples could not be tested because the residuals were too little. Details of the FilmArray positive samples are shown in Table 2.

**TABLE 2:** Results of the FilmArray respiratory panel

Test result	No of samples	No of samples tested PCR
Human Rhinovirus/Enterovirus	110	89
Human Rhinovirus/Enterovirus + Adenovirus	6	6
Human Rhinovirus/Enterovirus + Coronavirus HKU1	2	1
Human Rhinovirus/Enterovirus + Human Metapneumovirus	1	0
Human Rhinovirus/Enterovirus + Influenza A H3	1	1
Human Rhinovirus/Enterovirus + Parainfluenza Virus 1	1	1
Human Rhinovirus/Enterovirus + Parainfluenza Virus 3	5	5
Human Rhinovirus/Enterovirus + Parainfluenza Virus 4	1	1
Human Rhinovirus/Enterovirus + Respiratory Syncytial Virus	6	5
Human Rhinovirus/Enterovirus + SARS-CoV-2	2	2
Human Rhinovirus/Enterovirus + Adenovirus + Human Metapneumovirus	1	1
Human Rhinovirus/Enterovirus + SARS-CoV-2 + Adenovirus	1	1
Human Rhinovirus/Enterovirus + SARS-CoV-2 + Parainfluenza Virus 3	1	1
Human Rhinovirus/Enterovirus + Coronavirus OC43 + Parainfluenza Virus 3	1	1
Adenovirus	7	
Coronavirus HKU1	5	
Coronavirus NL63	3	
Coronavirus OC43	3	
Human Metapneumovirus	6	
Influenza A H3	5	
Parainfluenza Virus 1	7	
Parainfluenza Virus 3	6	
Respiratory Syncytial Virus	15	
SARS-CoV-2	20	
Adenovirus + Human Metapneumovirus	2	
Adenovirus + Parainfluenza Virus 1	1	
Adenovirus + Respiratory Syncytial Virus	2	
Adenovirus + Parainfluenza Virus 3 + Coronavirus NL63	1	
Coronavirus 229E + Coronavirus HKU1	1	
Coronavirus NL63 + Human Metapneumovirus	1	
Coronavirus NL63 + Respiratory Syncytial Virus	1	
Coronavirus OC43 + Parainfluenza Virus 3 + Respiratory Syncytial Virus	1	
Parainfluenza Virus 3 + Respiratory Syncytial Virus	2	
SARS-CoV-2 + Coronavirus HKU1	1	
SARS-CoV-2 + Parainfluenza Virus 1	1	
Total	230	115

### Primer design

To detect RVs and EVs in nasopharyngeal specimens with high sensitivity, we adopted real-time RT-nested PCR. We intended to design the first PCR for amplification of all viruses belonging to both RVs and EVs, and the second PCR for distinguishing RVs and EVs completely by using different primer sets. Primers suited for these requirements were searched by using viral sequences downloaded from GenBank. Table 3 shows the frequencies of mismatches between each of the selected primers and its corresponding region of 5,328 EVs and 1,627 RVs. The primers to specifically amplify both EVs and RVs, ERV1-F, ERV1-R, and ERV2-F, were highly matched with corresponding sequences of EVs and RVs: >2 mismatches are observed in very few cases (<0.2%). On the other hand, an RV-specific reverse primer RV2-R has >2 mismatches with 5,324 EV sequences (99.92%) and one RV sequence (0.06%); EV-specific reverse primer EV2-R has >2 mismatches with three EV sequences (0.06%) and 1,612 RV sequences (99.08%) in full-length primer sequences. When matching between primers and viral sequences was compared in five nucleotides from the 3ʹ end of primers, which have significant effect on PCR amplification (32), the specificity of these primers became more evident (Table 3). It should be noted that the fifth nucleotide from the 3ʹ end of primer RV2-R was set to thymidine instead of cytidine, the consensus nucleotide of RV sequences, to increase the number of mismatches against EV sequences by one at the expense of one mismatch with RV sequences.

**TABLE 3:** Numbers of EVs and RVs whose sequences have different numbers of mismatches with each primer sequence

Range	Number of mismatched bases	Number of viral sequences									
		Primer ERV1-F (17 nt)		Primer ERV1-R (22 nt)		Primer ERV2-F (18 nt)		Primer RV2-R (18 nt)		Primer EV2-R (18 nt)	
		EVs	RVs	EVs	RVs	EVs	RVs	EVs	RVs	EVs	RVs
In full-length primer	0	5,310	1,564	5,206	1,560	5,243	1,564	0	0	5,253	0
	1	13	63	108	66	80	63	1	843	66	0
	2	2	0	3	1	2	0	3	783	6	15
	3	1	0	2	0	0	0	2,287	1	1	212
	4	0	0	0	0	3	0	3,030	0	1	767
	5	0	0	1	0	0	0	5	0	1	591
	6	2	0	2	0	0	0	2	0	0	42
	7	0	0	2	0	0	0	0	0	0	0
	8	0	0	0	0	0	0	0	0	0	0
	9	0	0	4	0	0	0	0	0	0	0
In 3′-end five nucleotides	0	5,324	1,627	5,327	1,625	5,256	1,624	0	0	5,298	0
	1	3	0	1	2	71	3	2	1,627	27	0
	2	1	0	0	0	1	0	7	0	1	116
	3	0	0	0	0	0	0	2,325	0	2	1,178
	4	0	0	0	0	0	0	2,991	0	0	333
	5	0	0	0	0	0	0	3	0	0	0
At 3′ end	0	5,324	1,627	5,327	1,627	5,327	1,627	13	1,327	5,320	1,327
	1	3	0	0	0	0	0	5,314	0	7	0

### Testing of clinical specimens

**Fig 1:**
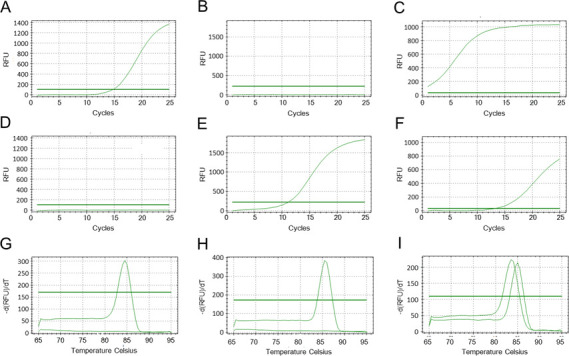
Real-time PCR after the first PCR for detection and discrimination of RV and EVs using SYBR green. Panels **A**, **B**, and **C** are amplification curves of clinical samples of RV, EV, and their mixture, respectively, measured using RV-specific primer RV2R. Panels **D**, **E**, and **F** are amplification curves of clinical samples of RT, EV, and their mixture, respectively, measured using EV-specific primers of EV2RA and EV2RB. Panels **G**, **H**, and **I** are melting curves of amplified products of panels **A **and **D**, those of panels **B** and **E**, and those of panels **C** and **F**, respectively.

We used the PCR assay for 115 nasopharyngeal swab specimens that have tested positive for Human Rhinovirus/Enterovirus by the FilmArray. The results showed that 103 specimens tested positive for RV-specific primer, seven specimens tested positive for EV-specific primer, four specimens tested positive for both RV-specific primer and EV-specific primer, and one specimen tested negative for either RV or EV primer. Typical amplification curves and melting curves in real-time PCR with SYBR Green using either the RV-specific primer or the EV-specific primer are shown in Fig. 1. Amplification of RV and EV sequences was distinguished by melting temperature of their amplicon. It was clearly demonstrated whether the specimen tested RV-positive, EV-positive, or both positive.

To examine the fidelity of the present assay, we identified the species and serotype of viruses in 114 RV- and/or EV-positive clinical samples by analyzing sequences of the second-PCR amplicons using BLAST (Table 4). Among them, 103 samples shown to be RV by PCR included sequences of either RV A, B, or C species; seven samples shown to be EV by PCR included sequences of either EV A or D species; four samples shown to be both RV and EV by PCR included both RV and EV species (RV A22/EV D68, RV A36/Cox A7, RV A56/Cox A6, and RV C2/Cox A4). These results were completely consistent with the results of the current PCR assay.

**TABLE 4:** Comparison between BLAST and PCR results

Blast results			PCR results
Species	Serotype	Number	
RV A	A1^*a*^	4	RV
RV A	A7	1	RV
RV A	A10	1	RV
RV A	A12	1	RV
RV A	A19	3	RV
RV A	A20	1	RV
RV A	A22	4	RV
RV A	A24	2	RV
RV A	A28	5	RV
RV A	A34	3	RV
RV A	A36	4	RV
RV A	A40	1	RV
RV A	A47	1	RV
RV A	A49	1	RV
RV A	A53	1	RV
RV A	A56	6	RV
RV A	A80	1	RV
RV A	A85	1	RV
RV A	A89	1	RV
RV A	A101	5	RV
RV B	B6	2	RV
RV B	B27	1	RV
RV B	B48	1	RV
RV C	C2	3	RV
RV C	C5	2	RV
RV C	C6	2	RV
RV C	C7	4	RV
RV C	C9	1	RV
RV C	C11	1	RV
RV C	C13	2	RV
RV C	C17	3	RV
RV C	C18	2	RV
RV C	C23	9	RV
RV C	C26	2	RV
RV C	C30	7	RV
RV C	C35	3	RV
RV C	C36	2	RV
RV C	C42	1	RV
RV C	C44	1	RV
RV C	C53	1	RV
RV C	C56	1	RV
RV C	C	5	RV
EV A	Cox^*b*^ A4	1	EV
EV A	Cox A6	1	EV
EV D	D68	5	EV
Dual	RV A22 and EV D68	1	RV and EV
Dual	RV A36 and Cox A7	1	RV and EV
Dual	RV A56 and Cox A6	1	RV and EV
Dual	RV C2 and Cox A4	1	RV and EV

^*a*^ A term of RV was omitted from names of enterovirus serotypes.

^*b*^ An abbreviation of Coxsackie.

### Clinical association

Characteristics and clinical presentations only between patients with RV A and RV C were compared because of a small number of those with RV B (Table 5). There was no significant difference between them.

**TABLE 5:** Characteristics and clinical presentations of RV A and RV C

	RV A	RV C	*P*-value^*a*^
Number of patients	25	33	
Age, median [IQR], year	2.06 [1.38, 3.47]	2.02 [1.30, 3.58]	0.956
Male sex (%)	18 (72.0)	17 (51.5)	0.175
Any underlying disease (%)	14 (56.0)	25 (75.8)	0.159
Previous history of asthma (%)	0 (0.0)	4 (12.5)	0.113
Asthma attack (%)	3 (12.0)	9 (27.3)	0.201
Oxygen demand (%)	6 (24.0)	12 (36.4)	0.396
Admission in the intensive care unit (%)	3 (12.0)	2 (6.1)	0.643

^*a*^ Between-group differences were tested by using Fischer’s exact test or Mann-Whitney *U* test.

## DISCUSSION

In this study, we reported a real-time nested RT-PCR assay with SYBR Green for detection and differentiation of RVs and EVs. There have been several studies to report real-time PCR assays for specific detection of RVs and EVs (23, 33–35). These studies, however, did not distinguish RVs and EVs successfully except for Österback et al. (23). They used locked nucleic acid probes specific for RVs and EVs to improve the specificity of the assay. Limitations of their method are low sensitivity due to a single-round PCR and possible cross-reaction of the probe with RVs and EVs (23).

During the COVID-19 pandemic, the FilmArray Respiratory Panel 2.1 became very popular in Japan because it can simultaneously detect multiple respiratory pathogens, including severe acute respiratory syndrome coronavirus 2 (SARS-CoV-2). In the Keio University Hospital, we have routinely tested pediatric patients with fever or respiratory symptoms by this panel since the pandemic. In the process of testing respiratory infections using the FilmArray, we encountered some problems. One of them is that RVs and EVs are not differentiated but diagnosed as “Human Rhinovirus/Enterovirus.” Therefore, the exact diagnosis of RV or EV infection was not obtained. This defect could make nationwide surveillance and epidemiologic studies difficult. It is also a problem that a dual infection of RV and EV cannot be detected. On the other hand, our assay does not have such problems. Furthermore, it serves to determine the genotype of RV and EV by sequencing and BLAST analysis of the final PCR product.

In the design of our assay, discrimination of RVs and EVs was achieved by difference in their specific primers. As shown in Table 3, RV-specific primer RV2-R and EV-specific primers EV2-RA/B have discriminatory numbers of mismatches with RV and EV sequences obtained from GenBank. The impact of mismatches between PCR primers and templates has been extensively studied (32, 36–38). Among them, Lefever et al. (32) investigated the effect using primers containing various numbers and locations of mismatches. Their results suggest that (i) a mismatch at the primer’s 3ʹ end, (ii) two or more mismatches in the last five nucleotides at the primer’s 3ʹ end, or (iii) four or more mismatches in a primer significantly reduce the efficiency of PCR amplification. When these rules are applied to predict the assay results using the primer sequences and the whole viral sequences collected, in 5,317 EVs, 16 (0.30%) would be undetected, and one (enterovirus D68) would be RV-positive; all of 1,627 RVs would be detected and diagnosed as RV. Although this study involved a small number of clinical samples that tested EV-positive, *in silico* analysis described as above suggests that the present assay will be able to discriminately determine early all EVs as well as RVs.

Among 115 FilmArray-positive samples, one sample was negative by our assay. It is unclear at present that this is due to a false positive by the FilmArray or a false negative of the present assay. Information on a nucleotide sequence of the amplified product on the FilmArray may be helpful for elucidating this kind of difference.

In this study, we found that rhinovirus A and C were the most common pathogens during the COVID-19 pandemic in pediatric patients in Keio University Hospital (Tables 2 and 4). This finding is in accordance with a previous report that rhinovirus A and C accounted for the majority of infections during the COVID-19 pandemic in neonates and young infants in Niigata, Japan (39). Further studies are required to see if such a trend has continued since the end of the COVID-19 pandemic.

There are several limitations in this study. First, although the assay was evaluated using more than 100 samples, larger-scale studies in more diverse populations may be required for validation. Second, the sensitivity of the present method was not assessed, although it is expected to be comparable to that of the FilmArray because the results of both assays were consistent. Third, adaptation of a nested PCR technique, enabling a higher sensitivity, can be troublesome in manipulation and also difficult for instrumentation.

In conclusion, we have developed a sensitive method capable of not only distinguishing between RVs and EVs but also detecting their co-infections. We hope that this system can be further developed and integrated into a tool for the simultaneous detection of other respiratory pathogens and serve as real-time monitoring for routine diagnosis and virus surveillance.
